# Wavelet-based analysis of ground deformation coupling satellite acquisitions (Sentinel-1, SMOS) and data from shallow and deep wells in Southwestern France

**DOI:** 10.1038/s41598-019-45302-z

**Published:** 2019-06-19

**Authors:** André Burnol, Hideo Aochi, Daniel Raucoules, Fernanda M. L. Veloso, Fifamè N. Koudogbo, Alfio Fumagalli, Pierre Chiquet, Christophe Maisons

**Affiliations:** 1 BRGM, BP 36009, 3 avenue Claude Guillemin, 45060 Orléans, France; 2 TRE-ALTAMIRA, Calle Còrsega 381-387, 08037 Barcelona, Spain; 3 TEREGA, 40 avenue de l’Europe, 64010 Pau, France; 4 MAGNITUDE, Centre Regain, Route de Marseille, 04220 Sainte-Tulle, France

**Keywords:** Hydrology, Natural hazards, Aerospace engineering

## Abstract

Acquisitions of the Sentinel-1 satellite are processed and comprehensively analyzed to investigate the ground displacement during a three-year period above a double gas storage site (Lussagnet and Izaute) in Southwestern France. Despite quite low vertical displacements (between 4 and 8 mm) compared to the noise level, the cyclic motion reflects the seasonal variations due to charge and discharge during summer and winter periods, respectively. We can simulate the ground deformation at both storage sites by a simple mechanical model. However, ground movements of low-magnitude may be also induced by natural factors, such as the temperature or the soil moisture. Using a wavelet-based analysis, we show there is a soil expansion in the Lussagnet zone that contrasts both in phase and period with the seasonal deformation and that is linked to the surface soil moisture measured by the SMOS satellite. This other displacement is consistent with the water infiltration in the unsaturated zone followed by the swelling of a clay layer. This work reveals the combination of two different processes driving the ground displacement with the same order of magnitude (about 6 mm), namely the pressure variation of a deep gas reservoir and the swelling/shrinking of the shallow subsurface.

## Introduction

The Underground Gas Storages (UGS) are designed to address different needs that include a strategic gas reserve, a regulation of the gas supply, meeting seasonal peak heating and electricity demand and balancing the intermittent supply of renewable energy. Gas is stored from spring to autumn when the demand is lower and withdrawn during the winter period from October to April, when the demand is higher. UGS development required an appropriate site selection based on subsurface characterization, a suitable performance analysis, based on a fully integrated geological, fluid-dynamic and geo-mechanical approach, and finally a monitoring over the entire life of the storage. An integrated monitoring should include a network of microseismic sensors, observation wells to follow the reservoir as well as overburden pressures and a technique to measure ground surface displacements over wide areas. Standard ground surface monitoring techniques provide information on a very limited number of points within an area, both in the cases of geotechnical monitoring (clinometers, extensometers…) or GPS. Allowing a higher density of measurement points, the ground monitoring using Differential Synthetic Aperture Radar interferometry (DInSAR) has been intensively developed in the last two decades^1,2^ and validated against ground-based measurements^3,4^. Land subsidence related to groundwater extraction or uplift caused by the recharge of aquifers of large cities are some applications of the DInSAR technique, e.g. the subsidence in Mexico^5,6^ or the uplift in Brussels^7^ and in the London administrative area^8^. Conversely, the monitoring of gas storage using DInSAR has received scarce attention in scientific literature^9,10^. A major limiting factor to this purpose was the non-availability of both spatially and temporally high-resolution Synthetic Aperture Radar (SAR) dataset. Indeed, it has been shown that DInSAR techniques are more performant on regular motions than on displacements that have non-linear behavior with respect to time (i.e with strongly varying rates) as such displacements require finer temporal sampling to be better characterized^4^. This last issue was particularly sensitive with several past space missions for which few acquisitions per year were available (e.g. about 10 per year for Envisat). That was a limitation on the precision on the characterization of an annual cyclic motion. The launch of the Sentinel-1 mission by the European Space Agency (ESA) changed drastically the availability of SAR data by regular acquisition^11^. The mission consists of two satellites, respectively Sentinel-1A launched in April 2014, followed by Sentinel-1B in April 2016. Combining Sentinel-1A/1B, SAR images are acquired in interferometric mode every 6 days since October 2016. Another limiting factor is the low amplitude of deformation associated to gas storage in deep geological layers, the displacement to be measured is in this case of the same order of DInSAR measurement precision (typically a few millimeters)^9,10^.

Due to the potential substantial damage to buildings and infrastructure, the quantification of the clay swelling potential of expansive soils is a major concern for prevention plans. In France, the shrink/swell risk is the second most important cause of financial compensation from insurance companies behind the flood risk. In 2010, the French Geological Survey (BRGM) published a 1:50 000 swelling-risk map of France. This map indexed the territory as (i) no, (ii) low, (iii) moderate, or (iv) high risk. In the Aquitaine Basin of interest for this study, there is a low or a moderate swelling risk. At this resolution, the heterogeneity of the mineralogical composition of the sedimentary formations is not considered^12–14^. Therefore, some predictions of this swelling-risk map may be locally inaccurate or even wrong, according to the lithological characteristics of sedimentary formation at the given location. One way to improve this map consists in monitoring the soil moisture variations and ground movements by the instrumentation of experimental sites^15^. Another way is to use remote sensing satellite or aerial photography. Until now, the use of DInSAR was not operational for the mapping of swelling clays, mainly because of the non-availability of both spatially and temporally high-resolution and high-quality SAR dataset suitable to the very high variability of such surface deformation phenomena. Twenty years later, the question “Can we map swelling clays with remote sensing?” asked by Van der Meer in 1999^16^ is still a topical issue.

This work investigates therefore two main questions: (1) Are the variations detected in DInSAR measurements strongly related to the underground gas storage operations at Lussagnet and Izaute sites?; (2) Can the DInSAR processing be used to assess locally the shrink/swell hazard as given by the 1:50 000 geological map? The results section of this paper is organized around these two questions.

## Data and Methods

### Geographical and Geological Setting

**Figure 1:**
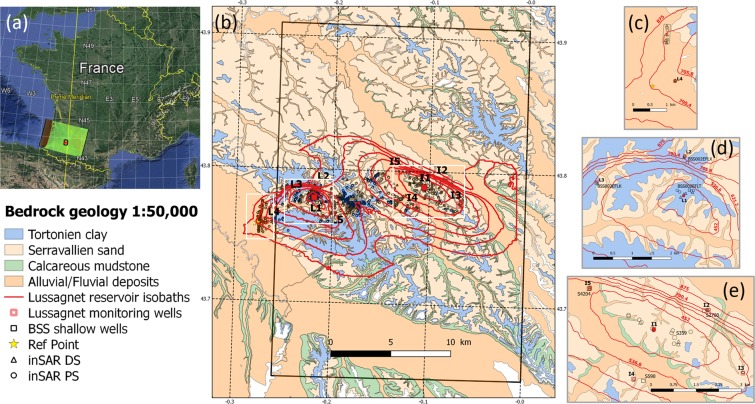
Location and geological map of the gas storage reservoir. (**a**) Regional setting in Southwestern France (Map data: Google, Landsat/Copernicus, SIO, NOAA, US Navy, NGA, GEBCO) that contains modified Copernicus Sentinel data (2016) and the 25 km cell of the EASE equal-area grid used by the SMOS satellite alongside the Prime Meridian (black square). (**b**) Simplified superficial geology of the studied zone modified from the BRGM geological map of France at the 1:50,000 scale showing the reservoir isobaths (red lines), the ~25 km SMOS grid cell and 1931 measurement points using SqueeSAR^®^ method including 1303 Persistent Scatterer PS (black circle) and 628 Distributed Scatterer DS (black triangle). Also shown are three selected zones (white empty rectangle) with the relative locations of the Lussagnet (L1) and Izaute (I1) gas exploitation wells (red filled square) and the Lussagnet/Izaute monitoring wells (red empty square). (**c**) Location of a reference zone (black rectangle) with four PS (black circle) and six DS (black triangle) and the Reference Point (yellow star) around the 790 m/NGF isobaths and outside the Tortonian clay surface layer. (**d**) Location of the Lussagnet exploitation zone with the selection of four PS and four DS inside the 452 m/NGF isobath and the location of two monitoring wells (L2 and L3). Also shown are the locations of shallow wells (black square) that are geological logs extracted from the BRGM subsurface database (BSS). (**e**) Location of the Izaute exploitation zone with eleven PS and three DS inside the 452 m/NGF isobaths, four monitoring wells (I2, I3, I4, I5) and three BSS shallow wells. Maps b-e were created using QGIS. QGIS Development Team, 2016. QGIS Geographic Information System (Version 2.18.0). Open Source Geospatial Foundation. http://qgis.org.

The studied area is a double storage site lying between both departments “Les Landes” and “Le Gers”, situated in the Aquitaine basin in Southwestern France, about 100 km north of the Pyrenees mountain (Fig. 1). The total reservoir structure is an anticline with two culminations, which are only approximately 10 km apart, the Lussagnet reservoir at the west side and the Izaute at the east. The highest point of the top of Lussagnet reservoir is located at a depth of about 550 m below ground level (mbgl) with a thickness of 40 m; the top of Izaute is at approximatively 510 mbgl^17^. Both reservoirs were deposited during the Eocen epoch, and are composed of unconsolidated sandstones, called “infra-mollassic sand”, with some interlayered claystones^18^. The hydrodynamic parameters of these reservoirs are variable. The total mean porosity of the sandstones varies from 20% to 35%, and their average permeability is from 1 to 10 Darcy. The Lussagnet and Izaute gas storage total capacity are respectively equal to 2.9 and 3.0 10^12^ m^3^ (referred to normal conditions) and only the working gas, respectively 1.4 and 1.5 10^12^ m^3^, is stored and withdrawn during the exploitation cycle. The remaining part, called cushion gas, supplies pressure support and prevents surface installations from excessive water production.

Concerning the surface layer, the shrink/swell hazard with 4 levels (null, low, moderate and high) has been evaluated in both departments “Les Landes” and “Le Gers” using different criteria, including the surface geological map at the scale 1:50 000 and other geotechnical and mineralogical criteria^19,20^. In particular, the Tortonien clay surface layer is characterized by a moderate shrink/swell hazard and the Serravallien sand surface layer by a low hazard. Just around both gas exploitation wells (L1 and I1), there is a moderate shrink/swell hazard at the Lussagnet site and a low hazard at the Izaute site (Fig. 1).

### Sentinel-1 DInSAR processing

SqueeSAR^®^ is a proprietary multi-interferogram technique which provides high precision measurements of ground displacement by processing multi-temporal satellite radar images acquired over the same area^21,22^. This technique allows the measurement of surface displacements by exploiting both point-wise coherent Permanent Scatterers (i.e. the PS) and partially coherent Distributed Scatterers (DS) (see Supplementary information for details). A dataset of 115 SAR images acquired by Sentinel-1A/B (C-band, IW mode) has been processed with the SqueeSAR^®^ technique providing 1931 measurement points including 1303 PS and 628 DS over the area of interest. The detected displacement is characterized by a time series sampled every 12 days until 1^st^ October 2016 and 6 days thereafter. The displacement measurements are carried out along the sensor’s Line of Sight (LOS), which is the sensor-to-target direction. DInSAR measures the projection of real motion along the LOS and provides 1D measurements. Those measurements are differential in space and time. They are spatially related to a reference point, and temporally to the date of the first available satellite acquisition. This reference point is steady through time and has been chosen around the 790 m/NGF isobath and outside the Tortonian clay surface layer (Fig. 1c). The combination of data results obtained from at least two different datasets with different acquisition geometries (ascending and descending), acquired over the same area in the same period, allows the estimation of 2D measurements, along the vertical and East-West directions. It is important to note that horizontal motion along the North-South direction cannot be measured with DInSAR techniques due to the low sensitivity to this component as this direction approximately corresponds to the flying direction of the satellites. This methodology requires that the same target is identified from both the ascending and the descending geometries. A projection of the LOS measurement along the vertical direction provides the real vertical displacement only in case the horizontal component of the movement is negligible. The random temporal changes on the surface of the Earth can reduce the signal-to-noise ratio (SNR), which is characterized through the interferometric coherence index given by the SqueeSAR^®^ technique.

### Wavelet-based analysis of DInSAR time series

DInSAR processing chains usually compute displacements as the superposition of linear and non-linear terms. Linear terms exhibit an infinite period along time, in the frequency domain. Non-linear component can exhibit different periods with higher frequency patterns at different time intervals, including seasonal fluctuations. The continuous wavelet transform (CWT) is especially suited to extract features from low signal-to-noise ratio time-series^23^. CWT expands time-series records into time/frequency space and can therefore identify localized intermittent periodicities^24^. The time-series input data must be equally spaced in time, i.e. evenly sampled with a fixed time interval. Additionally, two individual CWTs can be combined by using the cross wavelet transform (XWT) tool, if the relationship between two different time series is of interest. XWT is computed by multiplying the CWT of one time-series by the complex conjugate of the CWT of the second time-series. XWT image is the 2-D representation of the absolute value and the phase of the complex number in the time-frequency space. The absolute value of the XWT will be high in the time-frequency areas where both CWTs display high values, so this helps identify common time patterns in the two data sets. The phase of the XWT indicates the time lag between the two time-series. Consequently, this tool is very useful for exploring seasonal patterns which might have a time-lag (shown by the phase of the XWT) between the cause and the effect. XWT tool permits the recognition of common power and relative phase in time-frequency space, along with assessing confidence levels against red noise backgrounds^23^. For many geophysical phenomena, an appropriate background spectrum is either white noise (with a flat wavelet spectrum) or red noise (increasing power with decreasing frequency). These spectra are used to estimate the significance of a peak in the wavelet power spectrum. The proposed methodology here is to use CWT and XWT for which there are freely available Matlab codes (see Acknowledgements) to analyze comprehensively the DInSAR time-series vs. potential triggering factors time series (e.g. bottomhole pressure, rainfall, surface soil moisture, piezometric level). First, linear and non-linear components of the LOS displacement time series are separated: the linear component is computed by means of a linear least squares fitting and the non-linear component as the difference between the displacement time-series and the previously calculated linear component. Although SAR satellites have a regular revisit interval (6–12 days for Sentinel-1), some images may be missing or excluded from processing (Supplementary Fig. S2). After the separation between linear and non-linear components, the missing values of the non-linear component are linearly interpolated using a constant time interval of 12 days. Finally, we re-sample the time-series of potential triggering factors using the same time interval. For the bottomhole pressure or the piezometric level, we down-sample using the revisiting time period of Sentinel-1A (12 days) because they present generally a shorter time sampling (typically 1 day). For the rainfall, we calculate the accumulation during the Sentinel-1A period (12 days). For the surface soil moisture, we calculate an average value for each 12-day period using the Level 3 products of the Soil Moisture Ocean Salinity (SMOS) satellite as explained in the next section.

### Surface Soil Moisture extracted from SMOS Level 3 product

The first satellite mission to focus primarily on the collection of soil moisture data was the SMOS satellite (see Supplementary Information). We use here the term Surface Soil Moisture (SSM) to refer to the volumetric soil moisture in the first few centimeters (0–5 cm) of the soil. The SMOS Level 3 SSM products are downloaded through the website of the Centre Aval de Traitement des Données SMOS (CATDS, https://www.catds.fr/). The data are presented over the Equal-Area Scalable Earth (EASE grid 2)^25^ with a sampling of about 25 km x 25 km and the studied area is included in one grid cell (Fig. 1). The CATDS also provides a 10-day product that contains median, minimum and maximum values of soil moisture over 10 days. We use these 10-day SMOS-CATDS SSM products for descending overpasses between 18 October 2014 and 26 October 2017 to calculate an average value for each Sentinel-1 period (12 days).

## Results and Discussion

### LOS displacement observations around gas exploitations wells

**Figure 2:**
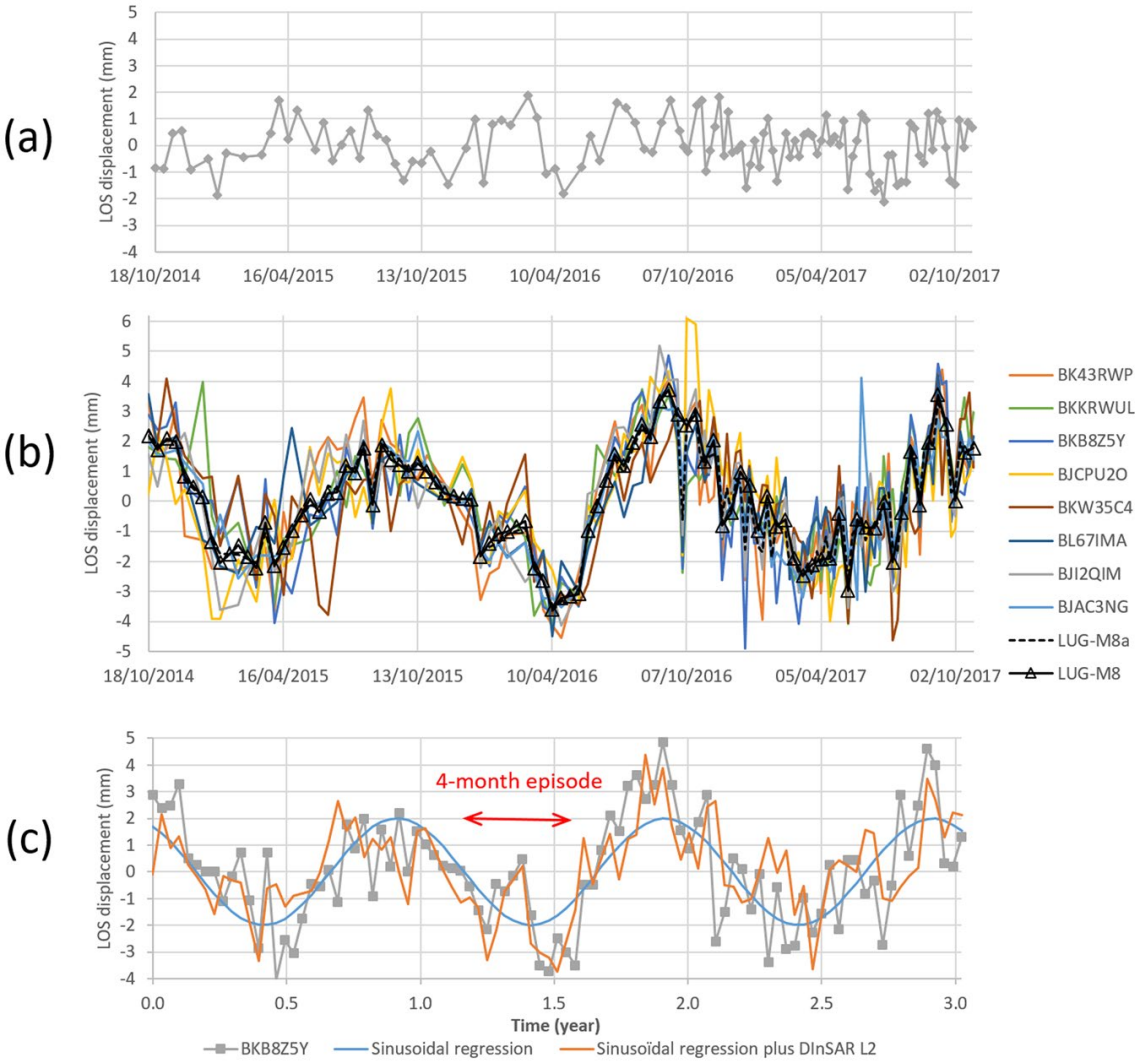
(**a**) Average LOS displacement in a reference zone away from L1 well around the 790 m/NGF isobath (see Fig. 1c). (**b**) Time profiles of eight Line of Sight (LOS) displacements (4 PS and 4 DS) around the Lussagnet exploitation wells and the mean value (called LUG-M8a) and the mean time value with a constant time sampling of 12 days (called LUG-M8). (**c**) Comparison of the time evolution of LOS displacement at BKKB8Z5Y point with the sinusoidal regression model and with the sum of the sinusoidal regression model and the DInSAR time series near L2 location.

In order to estimate the noise level in the DInSAR time series, we choose a reference zone far away from L1 and I1 wells around the 790 m/NGF isobath (Figs 1c and 2a). Supposing a sinusoid shape, the calculated noise level is about 2.66 mm (see Supplementary Information). That shows the power of DInSAR analyses to detect displacements in the studied zone with magnitudes of as small as a few millimeters.

**Figure 3:**
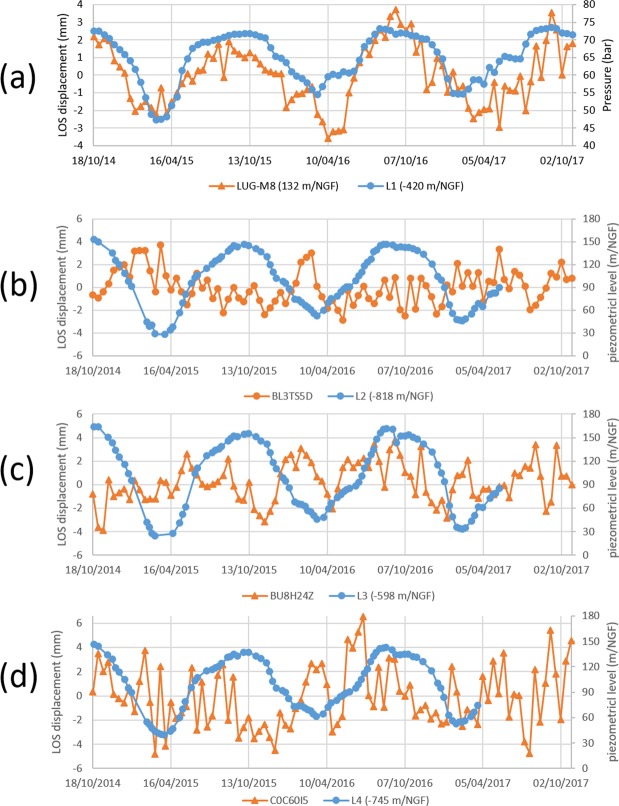
Comparison of average LOS displacement (LUG-M8) and bottomhole pressure at Lussagnet exploitation well location: (**a**) L1. Comparison of LOS displacement and piezometric level at three Lussagnet monitoring wells locations: (**b**) L2, (**c**) L3, (**d**) L4.

**Figure 4:**
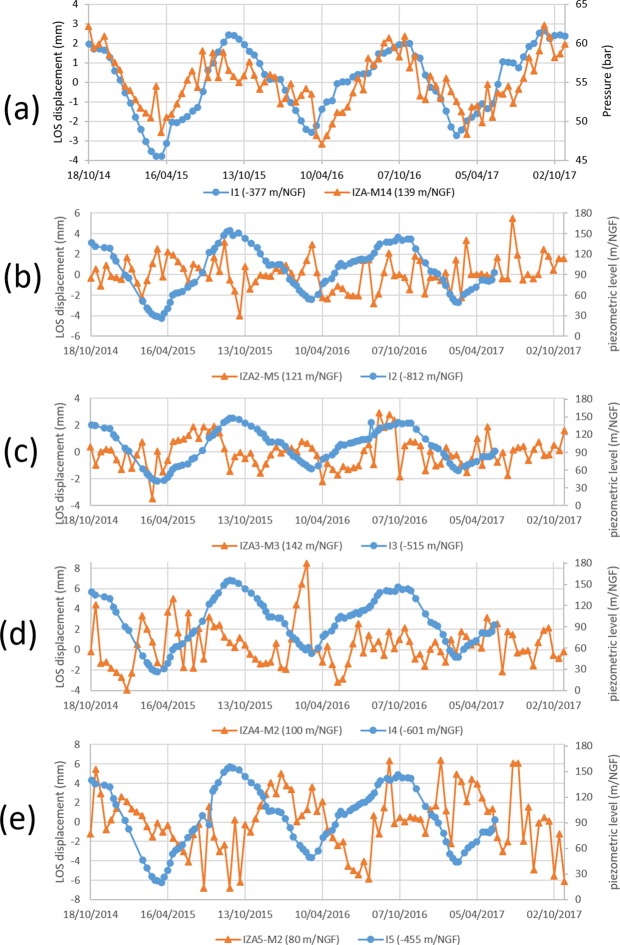
Comparison of average LOS displacement (IZA-M14) and bottomhole pressure at Izaute exploitation well location: (**a**) I1. Comparison of mean LOS displacement and piezometric level at four Izaute monitoring wells locations: (**b**) I2, (**c**) I3, (**d**) I4, (**e**) I5.

To investigate the DInSAR products in both gas exploitation zones, eight and fourteen DInSAR measurement points are respectively selected at Lussagnet site (4 PS and 4 DS) and Izaute site (11 PS and 3 DS) (Fig. 1d,e). This selection is based on three criteria: (i) the highest interferometric coherence index as calculated by the SqueeSAR^®^ technique (at least higher than 0.95); (ii) the proximity to the gas exploitation wells (less than 300 m from L1 and 1000 m from I1); (iii) a location outside buildings or surface infrastructures. We calculate the average of all the time-series using a constant time interval of 12 days (Fig. 2b). LUG-M8 and IZA-M14 time profiles are the average values of the LOS non-linear displacement in the Lussagnet and Izaute gas exploitation zone, respectively (see method section). During the three-year period, the average peak-to-peak LOS displacement is 5.8 mm ±1.3 mm in the Lussagnet case (Fig. 3a) and 5.1 mm ±0.6 mm in the Izaute case (Fig. 4a).

### Additive decomposition of the DInSAR time series using the reservoir pressure and the surface temperature

DInSAR time-series may be decomposed with the addition of a trend component T, a seasonal component S and an irregular residual component. In our case, the seasonal part S itself may be split into one bottomhole pressure component P and one surface temperature component ST (see Supplementary Information). The key result of this additive model is that the magnitude of the ST component is at least one order lower than the magnitude of P component (0.3 mm vs. 4 mm, see Supplementary Fig. S3).

### Vertical displacement due to the reservoir pressure variations

There is qualitatively a good correlation between the mean LOS displacement and the bottomhole pressure at both sites during the three-year period (Figs 3 and 4). The correlation coefficient is 0.77 in the Lussagnet case and 0.73 in the Izaute case. It is worth estimating a possible surface deformation due to the pressure variations in the reservoir and comparing quantitatively with the DInSAR surface observations^9^. In order to know briefly its amplitude, we adopt a linear elastic approach^26,27^. The predicted deformation pattern is quite independent, meaning that the deformation amplitude is close to zero at the middle of the two reservoirs (see Supplementary Fig. S4). During each injection/production cycle, the simulated vertical displacement attains the value of 8 mm at L1 well and 4 mm at I1 well. Around L1 and I1 wells, the simulated horizontal displacement is less than 2 mm (Supplementary Fig. S4e). This value is less than the estimated noise level in the reference zone (2.7 mm). Therefore, we can neglect the contribution of horizontal displacement around L1 and I1 wells and transform the displacement from LOS to the vertical using ${u}_{z}=LOS/\cos \,\theta$. Considering the local incidence angle of Sentinel-1 sensor (θ = 37.97°), we find a vertical displacement ${u}_{z}$ of 7.4 mm ±1.7 mm around the L1 well and 6.4 mm ±0.8 mm around the I1 well. This vertical displacement is fairly well estimated by the model in the Lussagnet case (8 mm) and lightly underestimated in the Izaute case (4 mm). Using a sensitivity analysis, we find that this simple model is consistent with DInSAR data around both gas exploitation wells (see Supplementary information).

**Figure 5:**
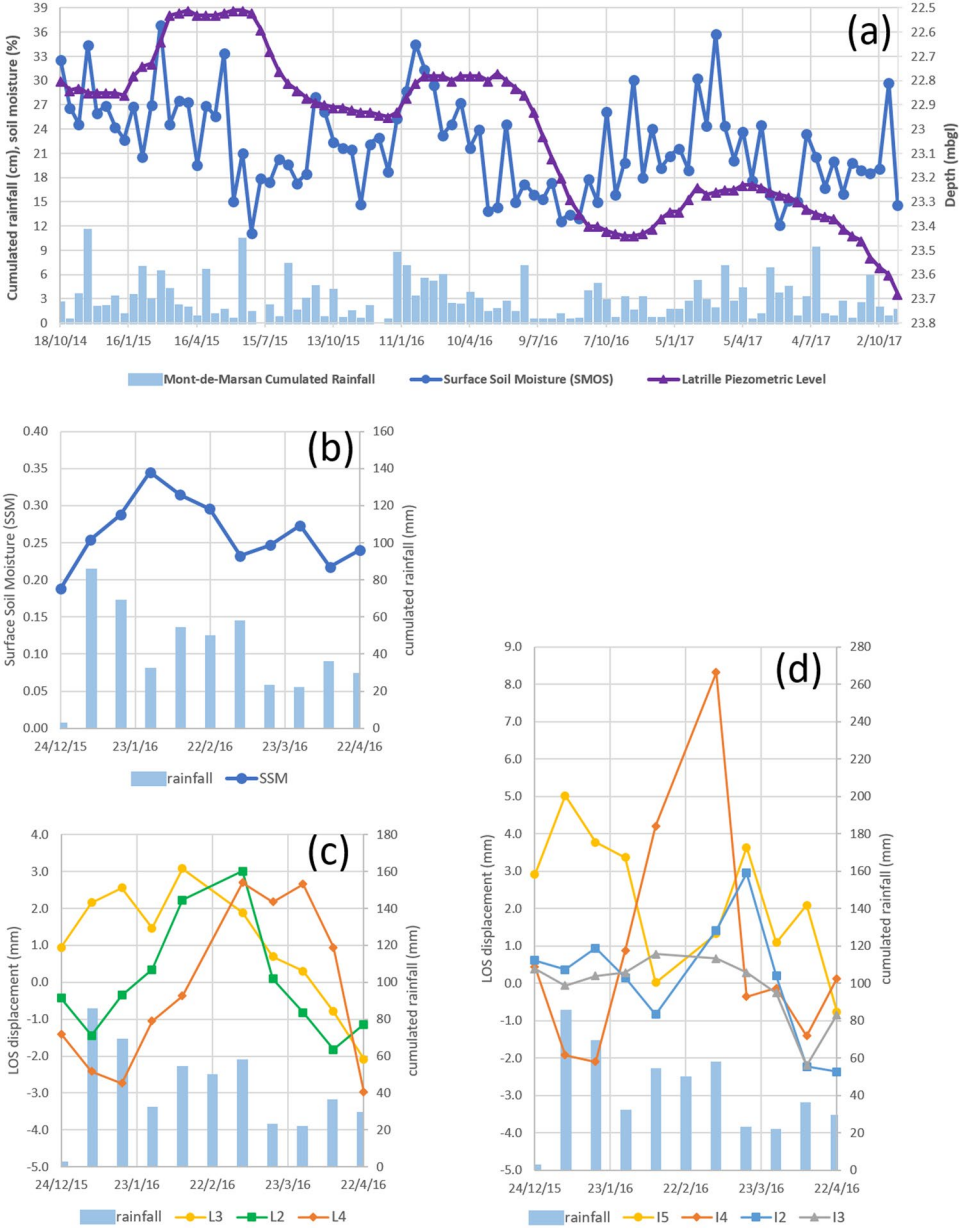
(**a**) Time series of three potential triggering factors: (1) rainfall accumulation at Mont-de-Marsan Weather station (during 12 days), (2) Surface Soil Moisture (SSM) at the 25 km SMOS grid cell around the studied area, (3) piezometric level at Latrille piezometer. In three panels below is shown the comparison during the 4-month episode (see text) between: (**b**) the rainfall and the SSM, (**c**) LOS displacements at L3, L2, L4 monitoring wells, (**d**) LOS displacements at I5, I4, I2, I3 monitoring wells.

Nonetheless, despite a general consistency, some features in the Lussagnet case can not be explained only by the reservoir pressure variations. In 2015, the maximum of pressure change in the reservoir was 24.6 bar with a minimum of LOS displacement of 4.1 mm, whereas in 2016 (Fig. 3a) the maximum of LOS displacement was 7.3 mm with a minimum of pressure change of 18.7 bar. More particularly, there is an average uplift of about 1.2 mm during a 2-month period during the first semester of year 2016, while there is a decrease of the reservoir pressure of 6 bar during the same period (Fig. 3a). Our hypothesis is that this difference is due to another process driving the ground deformation, namely the clay swelling in the Lussagnet zone (see the Tortonien clay layer in Fig. 1d). The vertical ground deformation due to the gas storage calculated by the mechanical model at the monitoring wells is less than the estimated noise level (2.7 mm) (Supplementary Fig. S4c). Therefore, it seems sound to investigate the LOS displacement at the monitoring wells and we compare first the LOS displacements at the monitoring wells and the piezometric levels (Figs 3 and 4). As predicted by the model, no obvious correlation is observed between these two time series. In order to extract features from these low signal-to-noise ratio time-series, we use now the wavelet-based analysis (see method section 3). The proposed methodology is to study the time-frequency relationships between the DInSAR displacements at the monitoring wells and three potential triggering factors (Fig. 5a and Supplementary Fig. S1): (i) the rainfall at the Mont-de-Marsan Weather Station; (ii) the soil Moisture acquired by the SMOS satellite in a 25 km grid cell around the studied area; (iii) the shallow phreatic groundwater piezometric level measured at Latrille.

### Wavelet-based analysis of LOS displacement at the monitoring wells in the Lussagnet zone

**Figure 6:**
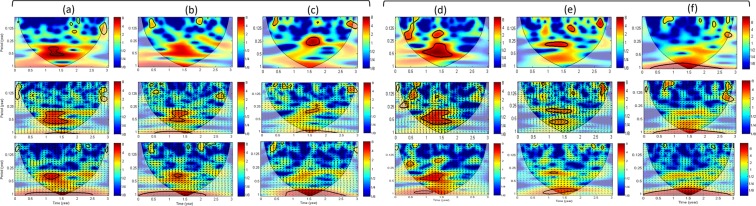
In the top, the continuous wavelet transform (CWT) of the mean LOS displacements is shown at 3 monitoring wells of Lussagnet and Izaute sites: (**a**) L3, (**b**) L2, (**c**) L4, (**d**) I4, (**e**) I2, (**f**) I5. The thick contour designates the 5 % significant level against red noise (see method section 3). The cone of influence (COI) where edge effects might distort the picture is shown as a lighter shadow. In the middle, the cross wavelet transform (XWT) of the cumulated rainfall and the LOS displacement at these six monitoring wells is shown. The relative phase relationship is shown as arrows, with in-phase pointing right and anti-phase pointing left and the rainfall leading by 90° pointing straight down. In the bottom, the same XWT transform is shown using the surface soil moisture instead of the rainfall.

From the analysis of the continuous wavelet transforms (CWT) of the LOS time-series (Fig. 6a), some power signals with a significant level against red noise can be recognized with a period of 4 to 8 months at L3 and L2 and with a period of about 4 months at L4 during the year 2016. CWT of the LOS time series at the three monitoring wells (L2, L3, L4) is now compared to the CWT of three natural triggering factors time series (Fig. 6). From XWT with the rainfall, there is a high common power with a significant level against red noise during the first semester of year 2016 with a period of 4 months and 8 months at L3 and L2 (Fig. 6a,b). A similar high common power with the rainfall can be recognized for the LOS displacement at L4 (Fig. 6c). During a 4-month episode, the rainfall is in phase with the LOS displacement at L3 (arrow pointing right) and is leading the LOS displacement at L2 (arrow pointing down). Using SSM instead of the rainfall, XWT gives a similar result with the period of 4 months at L3 and L2. Concerning the piezometric level at Latrille, there is no higher frequencies than the one-year cycle (Supplementary Fig. S5). In conclusion, the cross analysis with the rainfall and SSM reveal the role of a 4-month episode (between end of December 2015 and end of April 2016) on the ground deformation at the three monitoring wells (L3, L2, L4).

### Wavelet-based analysis of LOS displacement (LUG-M8) in the Lussagnet gas exploitation zone

**Figure 7:**
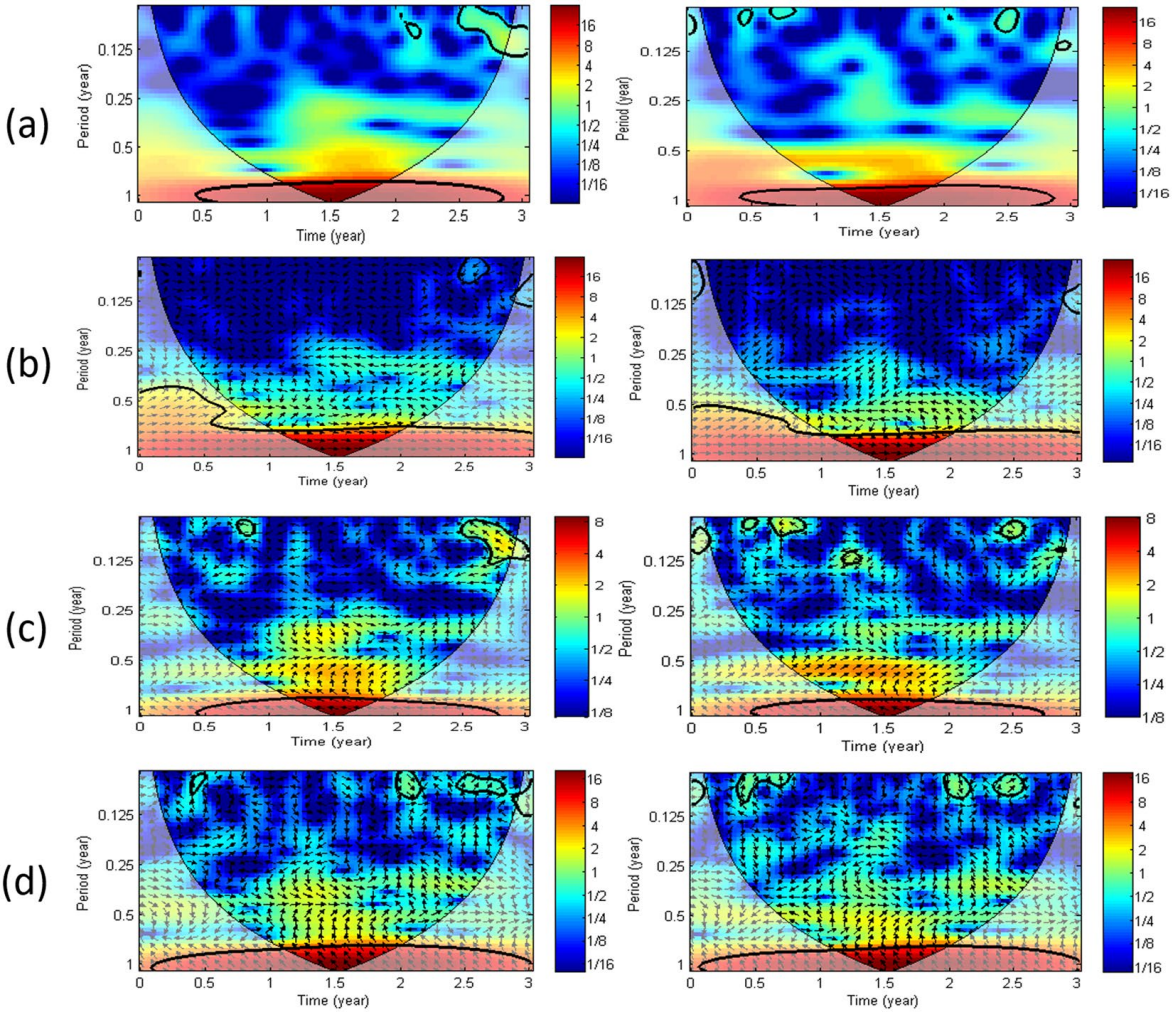
(**a**) Continuous wavelet transform (CWT) of the average LOS displacements is shown: on the left, around the Lussagnet injection well (LUG-M8) and on the right around the Izaute injection well (IZA-M14). (**b**) Cross wavelet transform (XWT) of the pressure in the deep reservoir and both average LOS displacements. (**c**) XWT transform of the cumulated rainfall and both average LOS displacements. (**d**) The same XWT transform is shown using the surface soil moisture instead of the rainfall. The relative phase relationship is shown as arrows, with in-phase pointing right and anti-phase pointing left and the first time series leading by 90° pointing straight down.

There is a clear 1-year cycle using the CWT of LUG-M8 (Fig. 7a) and a strong in-phase relation (arrows pointing right) with the bottomhole pressure using XWT (Fig. 7b). There is also a period of about 4 months but with a much lower magnitude than at the monitoring wells (Fig. 7a). Using XWT, there is the same common high power with the rainfall and SSM during the 4-month episode (Fig. 7c,d) and the rainfall is leading LUG-M8 (arrows pointing down).

### Modeling of the LOS displacement in the Lussagnet zone during the 4-month episode

**Figure 8:**
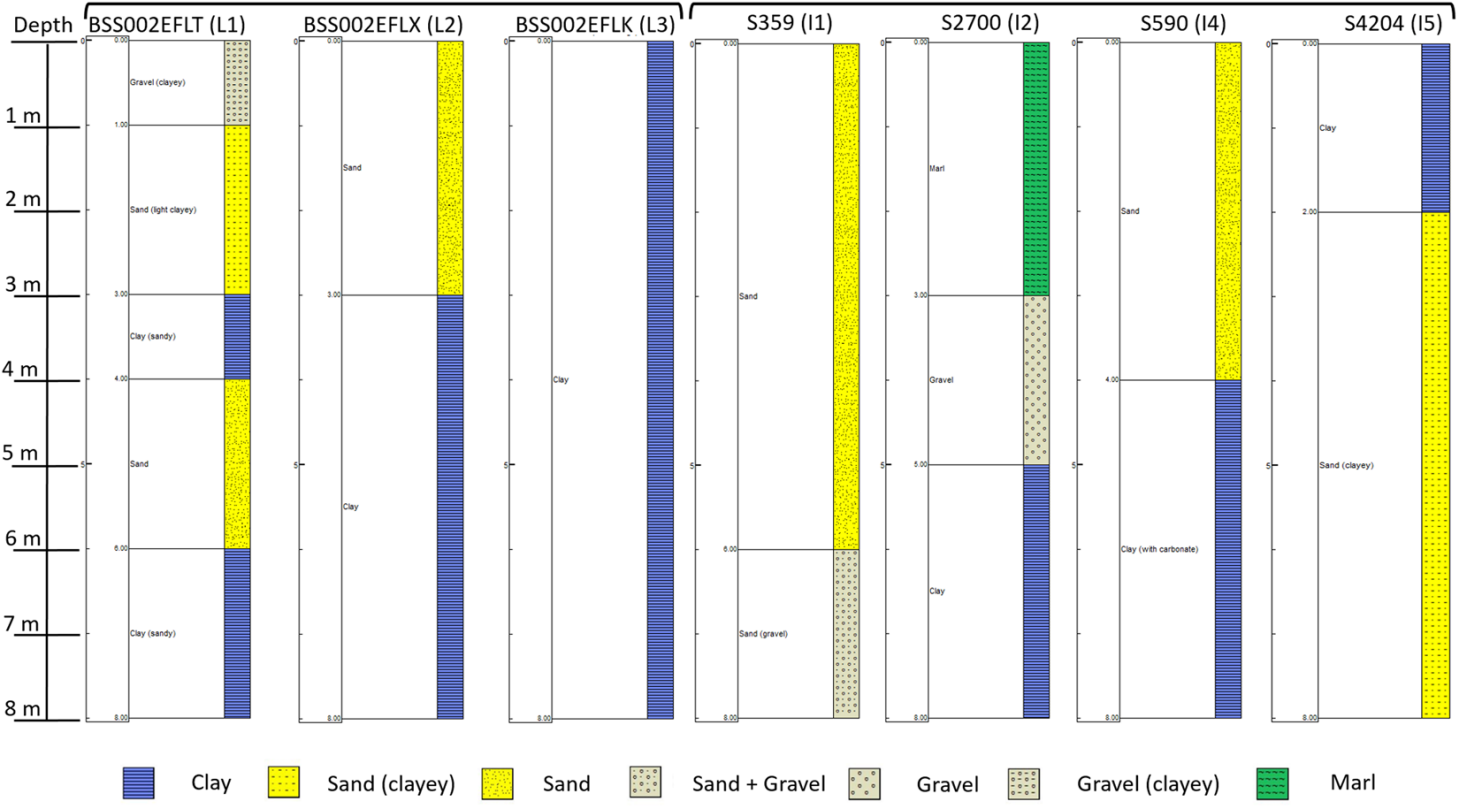
Geological Logs (see Fig. 1) extracted from the BRGM subsurface database (“Banque de données du sous-sol”, http://infoterre.brgm.fr/): around the Lussagnet exploitation well (L1) and two monitoring wells (L2, L3), around the Izaute exploitation well (I1) and three monitoring wells (I2, I4, I5).

We compare now in Fig. 5c the times series of the LOS displacement at the monitoring wells (L2, L3 and L4) with the rainfall and the SSM during the 4-month episode (between end of December 2015 and end of April 2016). The LOS displacement increase is 4.4 mm and 5.4 mm at L2 and L4, respectively, and the decrease is about 5 mm at L3. This LOS displacement is in phase with SSM at L3 and is leading the LOS displacement at L2 and L4. A possible explanation is that the observed soil expansion is due to the clay swelling and that the time lag is due the infiltration time of water in the unsaturated zone from the surface to the clay layer. Using the available geological logs from the BRGM subsurface database (BSS), we find indeed there is a clay layer at the surface at L3 and at the same depth (3 m) at L2 and L1 (Fig. 8). We try now to simulate the LOS displacement near L1 (called BKB8Z5Y) supposing that the displacement due to the clay swelling is the same at L1 and L2 (because of the same depth of the clay layer). We test a sinusoidal regression model SR(t) with a one-year period to simulate the seasonal displacement due to the gas exploitation:

1 $$SR(t)={A}_{max}\,\cos ((t+\phi )\times 2\pi /T)$$

where t is the time in days (relative to 18 October 2014), A_max_ the amplitude (2 ± 0.32 mm), φ the phase delay (32 days) and T the period (365 days). We calculate another model by adding to SR(t) the LOS displacement at L2:

2 $$M(t)=SR(t)+L2(t)$$

This second model fits indeed quite well the LOS displacement at L1 during the 4-month episode (Fig. 2c). In conclusion, the swelling/shrinking cycle takes about 4 months with a LOS displacement of about 5 mm, corresponding to a vertical displacement of about 6 mm (if we neglect the horizontal displacement). To support this conclusion, we use now the same wavelet-based method to analyze the LOS displacement in the Izaute zone.

### Wavelet-based analysis of LOS displacement in the Izaute zone

Using CWT, some power signals during the first semester of the year 2016 with a significant level against red noise can be recognized with a period of 4 and 8 months at I4 (Fig. 6d), with a period of 4 months at I2 (Fig. 6e) and with a period of 12 months at I5 (Fig. 6f). From the cross analysis with the rainfall and the SSM, we find a high common power during the first semester of year 2016 with a significant level against red noise with a period of 4 months at I4 and I2 (Fig. 6d,e). During this episode, the rainfall is leading the LOS displacement at I4 by about one month (arrow pointing straight down, Fig. 6d) and is in anti-phase with the LOS displacement at I2 (arrow pointing left, Fig. 6e) and also at I5 but with a much lower magnitude (Fig. 6f). From the analysis of the CWT of the InSAR time-series, we can observe a clear 1-year cycle (12-month period) for the Izaute exploitation zone (IZA-M14) during the three years (Fig. 7a). Interestingly, XWT reveals that the rainfall is also leading during this episode the LOS displacement IZA-M14 but with a very low magnitude compared to the Lussagnet case (Fig. 7c). The presence of small-scale lenses of clays in the Izaute exploitation zone (not indicated by the 1:50 000 geological map) may explain this last result. In conclusion, XWT with the rainfall and the SSM reveals the role of a 4-month episode for the LOS displacement around two monitoring wells in the Izaute zone (I4 and I2).

### LOS displacement during the 4-month episode in the Izaute zone

We compare now the time series of the LOS displacements near the Izaute monitoring wells during this 4-month episode. The LOS displacement at I5 is leading the LOS displacement at I4, which is leading the LOS displacement at I2 (Fig. 5d). There is also another uplift at I5 corresponding to the uplift at I2. There is no significant LOS displacement at I3. As indicated by the phase of the XWT (see previous section), the rainfall and the SSM is leading the LOS displacement at I4 by about one month and the LOS displacement at I2 by about two months. The LOS displacement at I4 displacement is very similar in phase with the LOS displacement at L2 (with a leading phase for L2). All these XWT results are consistent with the depth of the clay layer given by the geological logs from the BSS database (Fig. 8): 3 m at L2, 4 m at I4 and 5 m at I2. The complex behaviour at I5 (Fig. 5d) may be explained by the addition of one thin clay layer at the surface and another one at 5 m in the clayey sand (Fig. 8). The lack of ground deformation at I3 is consistent with its location in a sand formation (Fig. 1e).

### A new method coupling satellites acquisitions and the BSS database to improve the global French shrink/swell hazard map

Locating the areas of low-magnitude soil vertical expansion would be useful for predicting areas with a high risk of shrinking in the event of a long drought. The most complex task is to extract the signal linked to the clay swelling (typically 5 mm) from the noise in the DInSAR time series. The two limiting factors to this purpose is the quality of the SAR dataset (e.g. low quality due to a dense vegetation) and the availability of logs in the BSS near the DInSAR measurement points. Because of their influence on the soil moisture, the buildings should also be excluded when selecting the measurement points. We show here first that the wavelet-based analysis is a powerful tool to quantify the correlation (amplitude and phase) between the LOS displacement and the Surface Soil Moisture acquired using both satellites (Sentinel-1 and SMOS). As a second step, the geological logs extracted from the BSS database spatially close to DInSAR scatterers are used to test the phase consistency. This method may be generalized using a global SAR dataset to improve the French shrink/swell hazard map.

## Conclusion

By combining high temporal Sentinel-1 interferometric products (12 days) and observations from a network of shallow and deep wells, we performed a first integrated monitoring of a double gas storage site during three years. We observed a vertical ground deformation between 4 and 8 mm during the summer and winter periods due to the gas exploitation at both gas storages sites. A comprehensive analysis of the ground deformation at the Lussagnet site shows also a transient surface deformation that contrasts both in phase and period with the cyclic deformation due to gas exploitation, but with a similar vertical amplitude (about 6 mm). This last result is consistent with the swelling of a clay layer described in the geological logs extracted from the BSS database. In these terms, the high spatial-temporal resolution of the satellite Sentinel-1 reveals the combination of two different processes driving the ground displacement with the same order of magnitude, namely the pressure variation of a deep reservoir and the swelling/shrinking of the shallow subsurface. The process linked to the gas storage differs from the swelling/shrinking process in that it impacts a larger spatial scale (see Supplementary Fig. S4), hence it is homogeneous at the level of buildings or other surface infrastructures. This 3-year monitoring coupling satellites acquisitions and ground-based data is a first step and could be followed in the future by the aggregation of heterogeneous data over longer time periods. As a perspective of this work, the same method coupling both satellite acquisitions (Sentinel-1 and SMOS) and the BSS database can be generalised using a global SAR dataset to improve the French shrink/swell hazard map.

## Supplementary information

Supplementary Information file

## Data Availability

The datasets generated and/or analysed during the current study are available from the corresponding author on reasonable request.
