# Supplementary material for: Wavelet-based analysis of ground deformation coupling satellite acquisitions (Sentinel-1, SMOS) and data from shallow and deep wells in Southwestern France
Source: Sci Rep. 2019 Jun 19;9:8812. doi: 10.1038/s41598-019-45302-z (PMC6584567; doi:10.1038/s41598-019-45302-z)
Supplement: Supplementary file 1 — Supplementary Information file [file 41598_2019_45302_MOESM1_ESM.pdf]

SUPPLEMENTARY INFORMATION

Wavelet-based analysis of ground deformation coupling satellite acquisitions (Sentinel-1, SMOS) and data from shallow and deep wells in Southwestern France

André Burnol, Hideo Aochi, Daniel Raucoules, Fernanda M.L. Veloso, Fifamè Koudogbo, Alfio Fumagalli, Pierre Chiquet, Christophe Maisons

Prepared for Scientific Reports

Number of Pages: 12

Supplementary Methods, Results, Figures and Tables.

Number of Tables: 2

Number of Figures: 5

I/ Local setting of the studied zone showing the Mont-de-Marsan weather station and Latrille piezometer

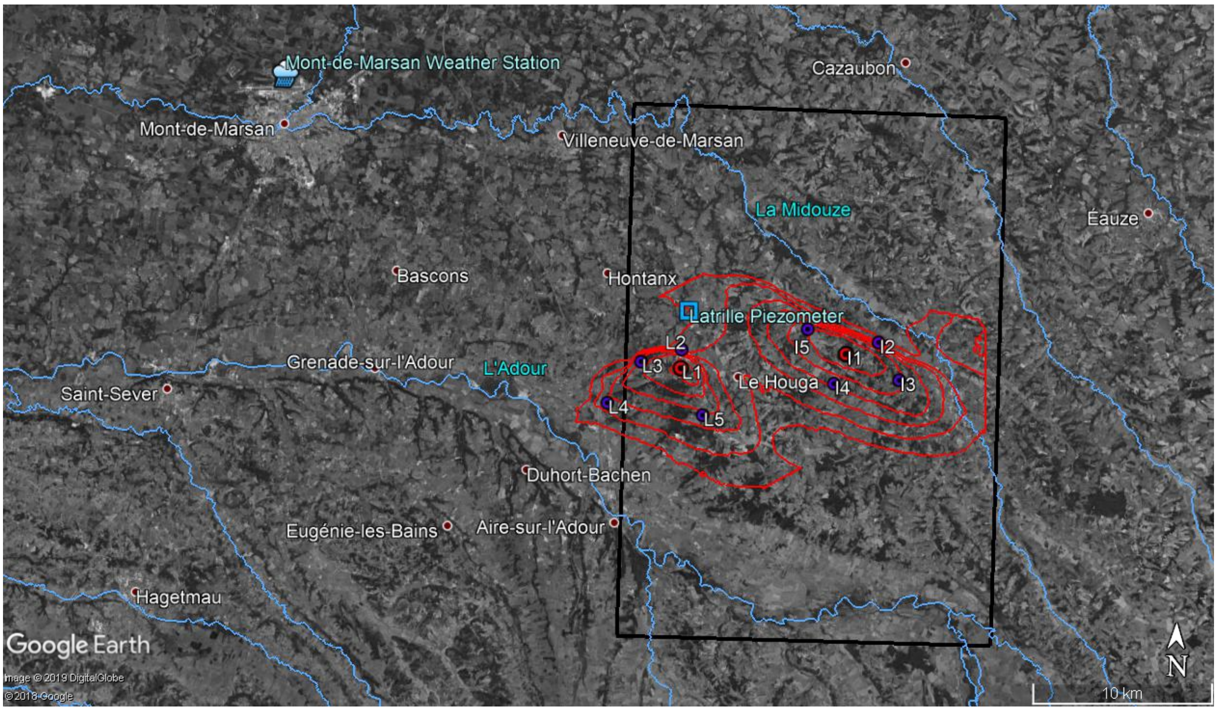

Fig. S1. Location map of the Mont-de-Marsan weather station and Latrille piezometer. The double gas storage reservoir isobaths between the river L'Adour at the West and La Midouze at the East are shown using red lines and the ~25 km SMOS cell is shown by a black square. On the western edge of Le Houga city is located the Lussagnet well L1 (red circle) and on the eastern edge the Izaute well I1 (red circle). The Latrille piezometric borehole and the Mont-de-Marsan weather station are located at a distance of about 3 km and 27 km from L1 well, respectively.

II/ Sentinel-1 satellite acquisitions using SqueeSAR® technique

Methods

SqueeSAR® is a second-generation PSInSAR algorithm, a method based on the processing of temporal series of co-registered SAR images acquired over the same target area. The main idea behind PSInSAR is to identify point-like targets (corresponding to single pixels or groups of a few pixels) exhibiting good phase coherence over the entire observation period via proper statistical analyses. Unlike the PSInSAR approach, the SqueeSAR® technique allows the measurement of surface displacements by exploiting both point-wise coherent scatterers (i.e. the PS) and partially coherent Distributed Scatterers (i.e. the DS).

The sensor to target direction is orthogonal to the orbit and it is called the Line of Sight (LOS). As any other PInSAR technique, SqueeSAR® measures the projection of the real displacement along the LOS, that is inclined with respect to the vertical axes. The main implications of the LOS measurements are the following:

- The same displacement can be measured with a different value from different acquisition geometries or satellites. Consequently, measurements obtained from different LOS cannot be directly compared.
- A re-projection of the LOS measurement along the vertical direction provides the real vertical displacement only in case the horizontal components of the movement are negligible.

During 2014/10/18-2016/10/1 period, images are acquired by Sentinel-1A every 6 days and during 2016/10/1-2017/10/26 period, images are acquired by Sentinel-1A or Sentinel-1B.

115 images are processed during this 3-year period and 9 images are missing or excluded from processing (see Table S1 and Fig. S2).

Table S1: SqueeSAR® parameters and results

**Track number**	8
**Geometry**	Descending
**# of images**	115
**Time period**	18/10/2014-26/10/2017
**Look angle θ (°)**	37.97°
**Azimuth angle δ (°)**	8.19°
**Number of measurement points (MP)**	1931
**Area**	87.5 km^2
**Measurement point density**	22.1 MP/ km^2
**Coordinate reference system**	NTF (Paris) Lambert Zone III
**Reference point**	X coordinate: 389548.84<br>Y coordinate: 3165408.00

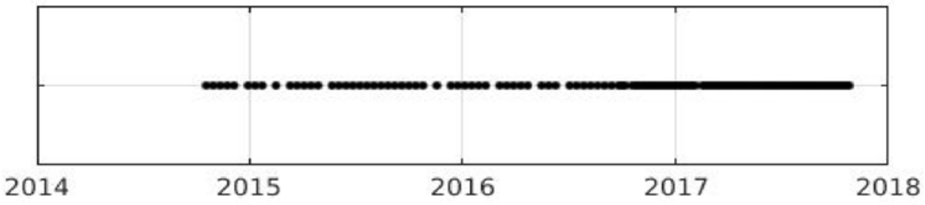

Fig. S2. Temporal distribution of Sentinel-1 acquisitions during the 3-year period

III/ Additive decomposition of LOS displacement using the reservoir pressure and the surface temperature

Methods

Time-series data are often governed by a trend component, a seasonal component and an irregular residual component. An additive model is appropriate if the magnitude of the seasonal fluctuations does not vary with the level of time series. Concerning the DInSAR time series, an additive form is assumed for the decomposition:

$$TS(t) = T(t) + S(t) + R(t) \qquad \text{(SE1)}$$

The trend part T is approximated by a second order polynomial function of time and the seasonal part S itself may be split into one bottomhole pressure component P and one surface temperature component ST:

$$S(t) = \alpha \times P(t) + \beta \times ST(t) \qquad \text{(SE2)}$$

Our additive decomposition model estimates therefore five coefficients, i.e. both coefficients (α, β) of seasonal part and three coefficients of the quadratic trend part, by minimizing the residual sum of squares (RSS) for all measurement points.

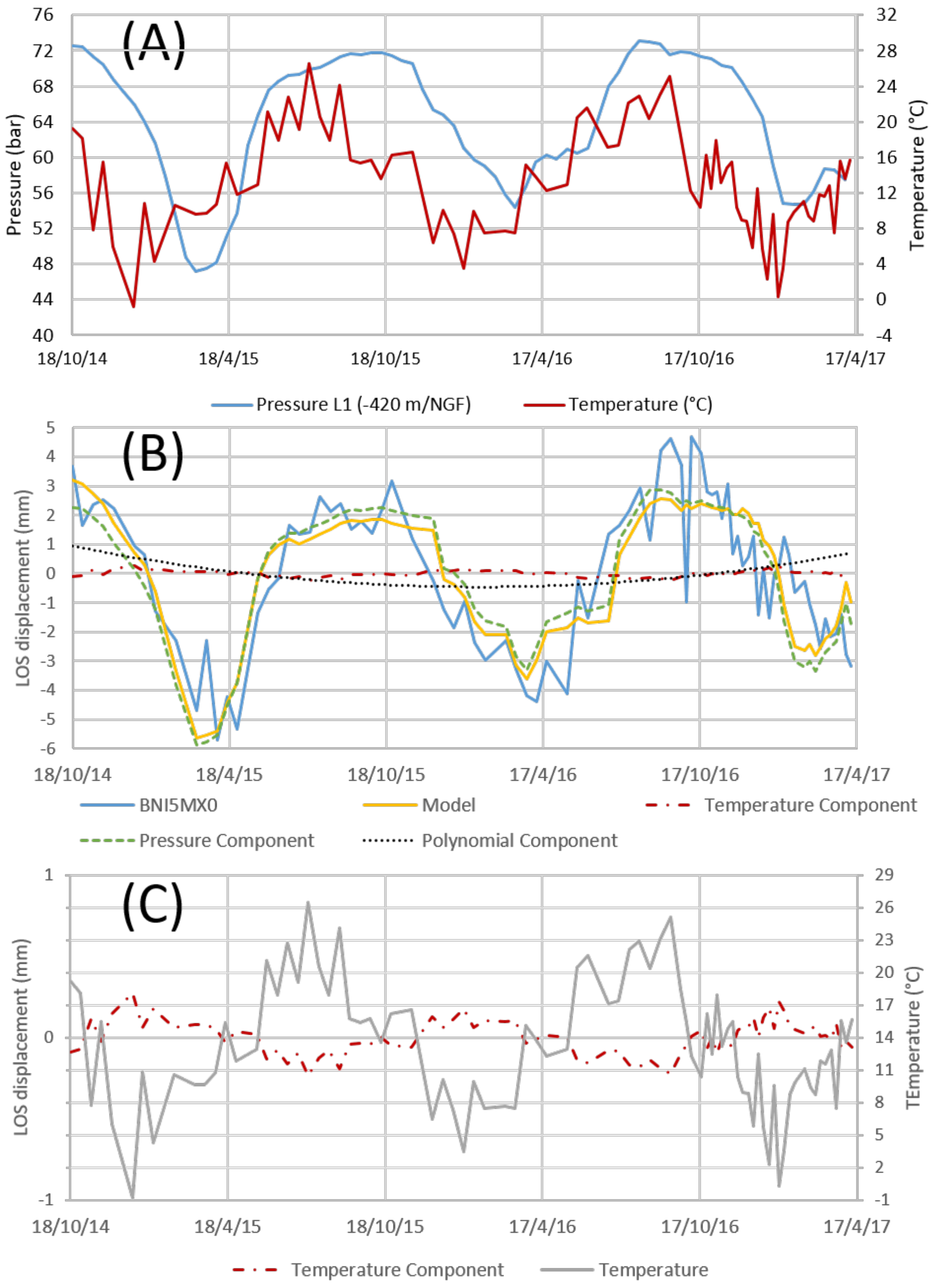

Fig. S3. Additive decomposition of the LOS displacement at the Lussagnet site using the reservoir pressure and the surface temperature. (A) Time profiles of the temperature from Mont-de-Marsan weather station and the gas pressure at Lussagnet well L1 during 2.5 years. (B) Additive decomposition of the LOS displacement near L1 (BNI5MX0). (C) Comparison of the temperature component of the LOS displacement at BNI5MX0 and the surface temperature. The displacement variation due to the temperature is in anti-phase with the temperature, while the pressure component is in phase with the bottomhole pressure.

IV/ Ground deformation modeling during each gas exploitation cycle

Methods

The vertical displacement (u_z) on the ground surface is expressed by an integral equation over the volume where pore pressure changes. For a uniform distribution of pressure change (ΔP) within a horizontally laid disk-shape reservoir (radius of R_0 and a depth between h_b and h_t) in a homogeneous, semi-infinite elastic medium, we have the integral equation given by Geertsma (1973):

$$u_z(r, z = 0) = -(1 - 2\nu)\frac{\alpha}{\mu}\Delta P \int_{h_1}^{h_2} \int_0^{R_0} R \int_0^{\infty} \xi J_0(R\xi) J_0(r\xi) e^{-h\xi} d\xi dR dh \qquad \text{(SE3)}$$

where cylindrical coordinate (r, z) is taken (Fig. S4a), and $J_0(x)$ is the Bessel function of the first kind for integer order 0. The horizontal component ($u_{x,y}$) is written by a similar integral equation as well. For taking into account of realistic reservoir geometry, we subdivide it by many point sources in order to conserve the reservoir volume and we take a summation over all the contribution of each part of the reservoir (Fig. S4b). The assumed model parameters are summarized in Table S2. This is a phenomenological, namely kinematic, expression without solving hydrogeological or geomechanical system of the reservoir. From the geological map, we estimate the extension of the reservoir as 5.4 km^2 for Lussagnet and 13.1 km^2 for Izaute, respectively. The gas is stored at the top of the reservoir such that the aquifer level is lowered. Schematically we simplify this by a vertical cylindrical reservoir in which the injected gas pushes down the native water. The modeling geometry is therefore simply vertical so that the deformation pattern calculated through Equation (SE3) concentrates around the reservoir surface (Fig. S4c-e). From this estimation, the vertical displacement on the ground surface arrives at 8 mm and 4 mm in the Lussagnet case and in the Izaute case, respectively (Fig. S4c). The deformation pattern is quite independent, meaning that the deformation amplitude is close to zero half way in-between the two reservoirs. As it is clear from Equation (SE3), the amplitude of deformation depends strongly on the material constants (ν, μ, α) given in Table S2. In the case where these three parameters vary with ±10%, u_z changes between +41% and -30%. In the Izaute case, this gives an upper bound of the simulated vertical displacement of 5.6 mm, which is consistent with the DInSAR measurement (see main text, 6.4 ± 0.8 mm). It can also be noted that a smaller reservoir geometry shall lead to a larger amplitude at the center of the reservoir.

The root mean square (RMS) of LOS displacement in the reference zone is 0.94 mm (Fig. 2a). Supposing a sinusoid shape, the peak-to-peak LOS displacement is $2\sqrt{2} \times 0.94 = 2.66$ mm. This value is our estimated noise level in the DInSAR time series in the studied zone.

Around the gas exploitation wells (L1 and I1), the predicted horizontal displacement is less than 2 mm (Fig. S4d-e). This value is less than the estimated noise level in the reference zone (2.7 mm). Therefore, we can neglect the contribution of horizontal displacement around L1 and I1 wells and transform the displacement from LOS to the vertical using $u_z = LOS/\cos\theta$.

Around the monitoring wells (L2-4 and I2-5), the predicted vertical displacement during each cycle is less than 2.5 mm (Fig. S4c). This value is less than the estimated noise level in the reference zone (2.7 mm). Therefore, we investigate by a wavelet-based analysis the LOS displacement at these monitoring wells to track another process driving the ground deformation in the studied zone.

Table S2: Model parameters

Parameter	Quantity and Unit
Poisson ratio $\boldsymbol{\nu}$	0.3
Medium rigidity $\boldsymbol{\mu}$	8 GPa
Biot's coefficient $\boldsymbol{\alpha}$	0.9
Lussagnet reservoir (surface S, top depth h_t, bottom depth h_b)	(5.4 km^2, 550 m, 715 m)
Pressure change ΔP in Lussagnet reservoir	20 bar
Izaute reservoir (surface S, top depth h_t, bottom depth h_b)	(13.1 km^2, 510 m, 590 m)
Pressure change ΔP in Izaute reservoir	15 bar

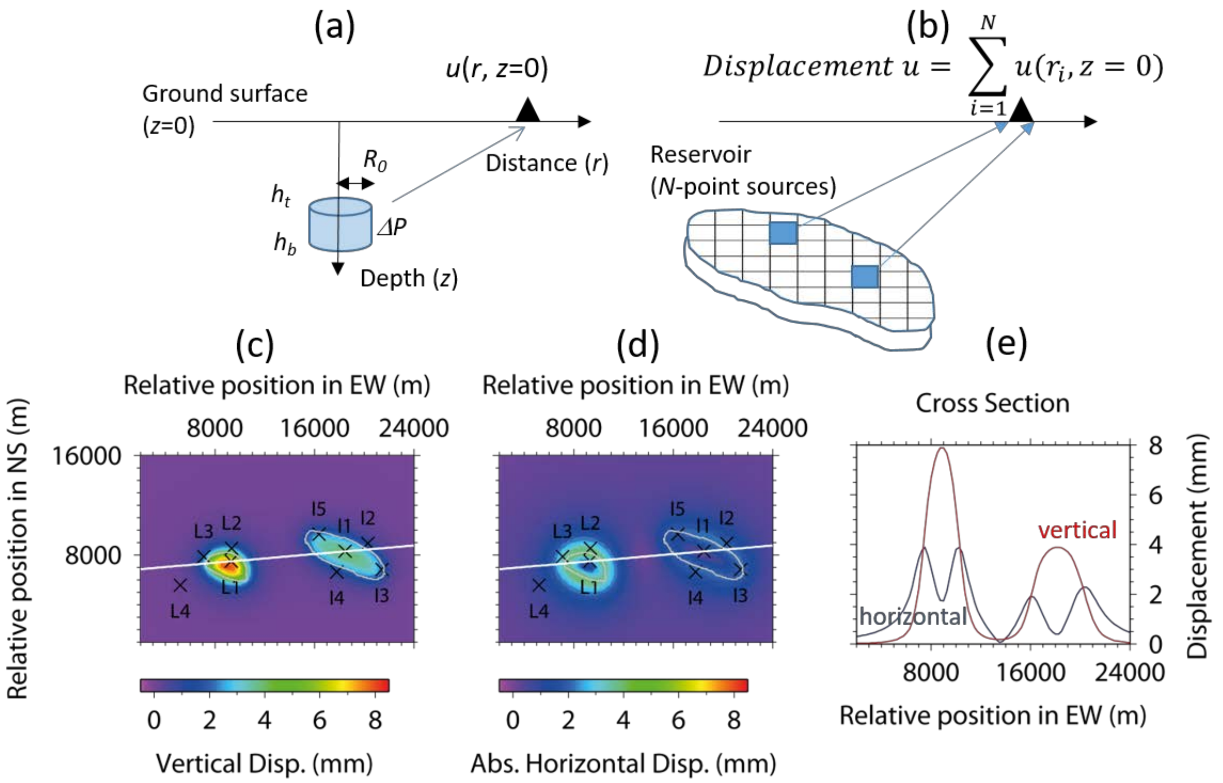

Fig. S4. (a) Geometry of each source cell of the reservoir (top depth h_t and bottom depth h_b) and pressure variations ΔP used by the model. (b) Geometry of the reservoir (N-point sources) and impact of each cell onto the ground displacement u. (c) Absolute value of the predicted vertical ground displacement (millimeter) during each injection/production simultaneous cycle at the Lussagnet and Izaute site. Also shown are the locations of the exploitation wells L1 and I1, the monitoring wells (L2, L3 and L4) and (I2, I3, I4, I5) at the Lussagnet and Izaute site. (d) Absolute value of the predicted horizontal ground displacement during each cycle. (e) Cross section along L1 and I1 (white line in panels c and d) gives the absolute value of the predicted vertical and horizontal displacements. Figures c-d-e generated with Generic Mapping Tools (GMT 5.1.2, http://gmt.soest.hawaii.edu).

V/ Surface Soil Moisture data extracted from SMOS level 3 products

Methods

SMOS satellite was successfully launched on the 2n^{d} of November 2009 by the European Space Agency (ESA). SMOS uses microwave radiometry for estimating soil moisture. We use here the term Surface Soil moisture (SSM) to refer to the volumetric soil moisture in the first few centimeters (0-5 cm) of the soil. L-band radiometry is achieved resulting in a ground resolution of 50 km. SMOS Levels L0 to L2 processing products are developed by the ESA. The products are divided in half orbits, from pole to pole, ascending or descending, spanning about 50 minutes of acquisition [1]. Level 3 products are geophysical variables with improved characteristics through temporal resampling or processing. In order to prevent any inconsistency resulting from interpolation over highly heterogeneous surfaces, no spatial averaging is operated in the algorithms. It must also be noted that ascending and descending overpasses are bound to show different values of the retrieved parameters that may not be always comparable and they are thus retrieved separately. The SMOS Level 3 SSM (RE04v300) products were downloaded through the website of the Centre Aval de Traitement des Données SMOS (CATDS, https://www.catds.fr/). The data are presented over the Equal-Area Scalable Earth (EASE grid 2) [2] with a sampling of about 25 km x 25 km (Fig. 1).

The performance of each satellite SSM product depends on many factors such as, but not limited to, soil type, climate, presence of noise (Radio Frequency Interference) and land cover. It is therefore difficult to predict the performance of SSM products over a region, without performing a quality assessment using in situ measurements on that region. A recent published evaluation of the accuracy of SMOS-CATDS products between 1 January 2016 and 30 June 2017 in southwestern France [3, 4] gives an average bias, RMSD (Root Mean Square Difference), ubRMSD (unbiased Root Mean Square Difference) of about -10 vol.%, 12 vol.% and 7 vol.%, respectively. The SMOS-CATDS products thus underestimate the surface soil moisture (with quite high negative bias values) but the ubRMSD of 7% is similar to the best values obtained by other satellites in Southwestern France (about 6%) [3, 4]. The filtered daily global maps are aggregated over different time periods. The SMOS revisit period is 3 days at the equator, so the Earth's surface is covered by SMOS field of view in 3 days and a 3-day global product with a 3-day moving window is provided by CATDS by aggregating the daily global maps. The retrieved SSM data are filtered using the Data Quality Index (DQX) provided in the product (DQX < 0.06 m3/m3). The filtering processor is then used over the Level 3 retrievals, to select the best estimation of soil moisture if several retrievals are available for a given day.

VI/ Continuous Wavelet Transform (CWT) of potential triggering factors of ground deformation

Results

From the analysis of the CWT of the rainfall time series (Fig. S5), we can observe two cycles (8-month and 4-month) during the year 2 in addition to the 1-year cycle. The CWT of the Surface Soil Moisture is similar to the rainfall CWT for the 4-month period, with a higher power for the 1-year cycle but with less power for the 8-month period. From the CWT analysis of the Latrille piezometric level, we can observe only the 1-year cycle and no patterns in higher frequencies.

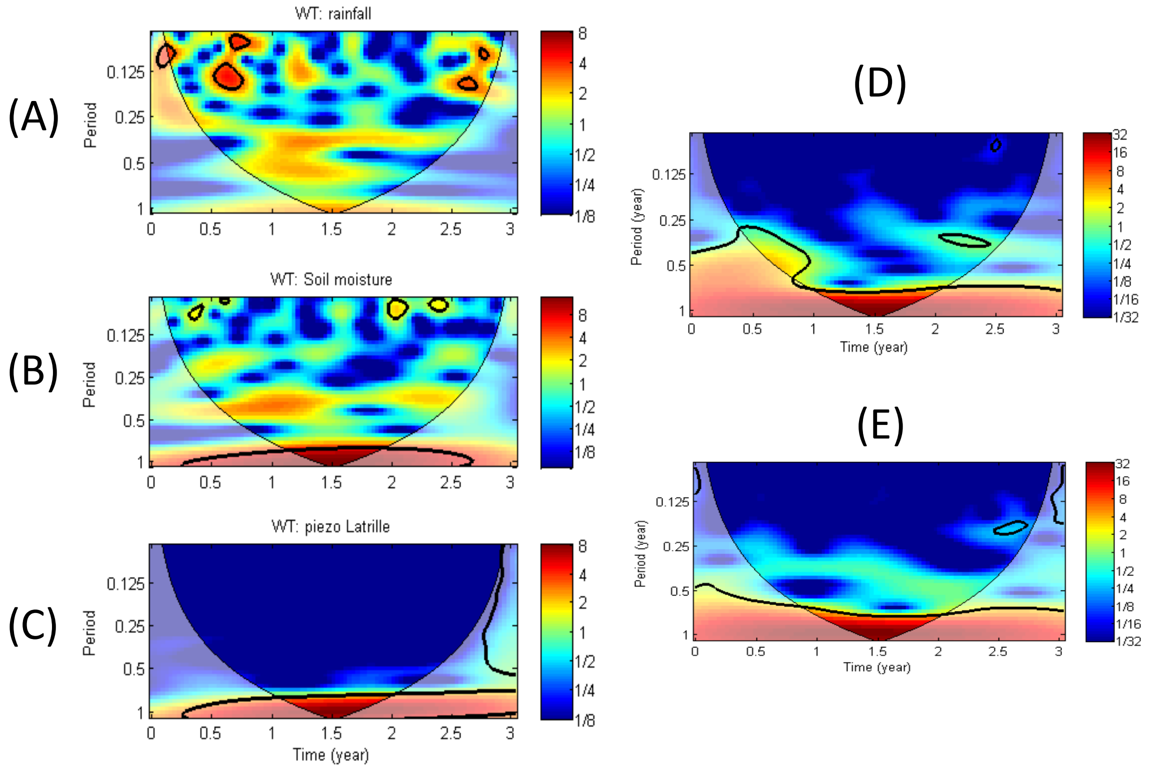

Fig. S5. Continuous wavelet transform (CWT) of potential triggering factors for ground displacement. (A) Accumulation of rainfall during 12 days at the Mont-de-Marsan weather station. (B) Surface soil moisture (SSM) observed by SMOS satellite in the ~25 km grid cell. (C) Piezometric level at Latrille station. (D) Lussagnet bottomhole reservoir pressure at L1 well. (E) Izaute bottomhole reservoir pressure at I1 well.

Supplementary references

1. Kerr, Y. H. *et al*. The SMOS Soil Moisture Retrieval Algorithm. *IEEE Transactions on Geoscience and Remote Sensing* **50**, 1384-1403 (2012).
2. Brodzik, M. J., Billingsley, B., Haran, T., Raup, B. & Savoie, M. H. EASE-Grid 2.0: Incremental but Significant Improvements for Earth-Gridded Data Sets. *ISPRS International Journal of Geo-Information* **1** (2012).
3. Al Bitar, A. *et al*. The global SMOS Level 3 daily soil moisture and brightness temperature maps. *Earth System Science Data* **9**, 293-315 (2017).
4. El Hajj, M. *et al*. Evaluation of SMOS, SMAP, ASCAT and Sentinel-1 Soil Moisture Products at Sites in Southwestern France. *Remote Sensing* **10** (2018).
